# Natural Bioproducts with Epigenetic Properties for Treating Cardiovascular Disorders

**DOI:** 10.3390/genes16050566

**Published:** 2025-05-10

**Authors:** Olaia Martínez-Iglesias, Vinogran Naidoo, Iván Carrera, Lola Corzo, Ramón Cacabelos

**Affiliations:** EuroEspes Biomedical Research Center, International Center of Neuroscience and Genomic Medicine, 15165 Bergondo, Corunna, Spain; neurociencias@euroespes.com (V.N.); biotecnologiasalud@ebiotec.com (I.C.); analisis@euroespes.com (L.C.); rcacabelos@euroespes.com (R.C.)

**Keywords:** cardiovascular disorders, epigenetic, bioproduct, epidrug

## Abstract

Cardiovascular disorders (CVDs) are the leading cause of mortality worldwide, highlighting an urgent need for innovative therapeutic strategies. Recent advancements highlight the potential of naturally derived bioproducts with epigenetic properties to offer protection against CVDs. These compounds act on key epigenetic mechanisms, DNA methylation, histone modifications, and non-coding RNA regulation to modulate gene expression essential for cardiovascular health. This review explores the effects of various bioproducts, such as polyphenols, flavonoids, and other natural extracts, on these epigenetic modifications and their potential benefits in preventing and managing CVDs. We discuss recent discoveries and clinical applications, providing insights into the epigenetic regulatory mechanisms of these compounds as potential epidrugs, naturally derived agents with promising therapeutic prospects in epigenetic therapy for CVDs.

## 1. Introduction

Cardiovascular disorders (CVDs) represent the leading cause of mortality and a significant health challenge worldwide, accounting for approximately 17.9 million deaths annually. This number is projected to increase to over 23.8 million by 2030, signifying a growing global health crisis. The prevalence of heart disease has surged dramatically, from 271 million cases in 1990 to 523 million in 2019, accompanied by a corresponding increase in disability-adjusted life years from 17.7 million to 34.4 million within the same period. In the United States alone, nearly half of all adults are affected by some form of heart disease, contributing to 25–30% of deaths each year in developed nations. The economic impact is profound, with the costs associated with managing heart disease and stroke exceeding USD 1.1 trillion annually, emphasizing the urgent need for more effective management strategies.

The increasing prevalence of cardiovascular diseases is further exacerbated by population aging and unhealthy lifestyle factors, including sedentary behavior, poor dietary habits, and smoking, which significantly contribute to higher morbidity and mortality rates worldwide [1,2]. The mean doubling time for CVD mortality is approximately seven years, with mortality rates in older populations being dozens to hundreds of times higher than in younger adults [2]. Unfavorable lifestyle behaviors are associated with a twofold or greater increase in risk of all-cause mortality, CVD mortality, myocardial infarction, and stroke [3].

Given these challenges, developing new strategies to treat and prevent cardiovascular diseases is critical [4]. Prevention approaches include primordial prevention, which aims to prevent the development of risk factors, and primary prevention, which targets individuals with existing risk factors before disease manifestation. These strategies complement the treatment of established diseases and are essential for reducing the global burden of CVD [4]. Despite extensive research, epigenetic approaches are still not routinely used in cardiovascular treatment [5]. Less than 5% of current therapies target epigenetic mechanisms, highlighting a significant opportunity for novel therapeutic development [6,7]. Emerging evidence suggests that natural compounds with epigenetic properties could fill this gap by providing effective, well-tolerated options for preventing and treating CVD [8]. Understanding the relationships among genetic, epigenetic, and environmental factors is crucial for addressing the complexity of heart diseases such as atherosclerosis, hypercholesterolemia, valvular diseases, heart failure, hypertensive syndromes, and other blood vessel disorders [1,2].

Epigenetics, the study of heritable changes in gene function that do not involve changes in the DNA sequence [9], has emerged as a significant field of research offering potential breakthroughs in understanding and treating CVD. Epigenetic disruptions, including aberrations in DNA methylation, abnormal chromatin remodeling, histone modifications, and dysregulated non-coding RNA, are increasingly recognized as critical mechanisms in the pathogenesis of various CVDs [10]. DNA methylation typically occurs at CpG dinucleotides and involves adding a methyl group to the cytosine ring. This modification can silence gene expression and is crucial in cardiovascular health. For example, altered endothelial nitric oxide synthase (*eNOS*) gene promoter methylation patterns are linked to endothelial dysfunction, a precursor to atherosclerosis. Histone modifications, including acetylation and methylation, affect chromatin structure and gene accessibility. Histone acetylation, which generally increases transcription, can be dysregulated in CVD, affecting genes involved in inflammatory responses and cardiac hypertrophy. Non-coding RNAs, such as microRNAs and long non-coding RNAs, regulate gene expression at the post-transcriptional level. MicroRNAs such as miR-133 and miR-1 are involved in cardiac pathology by modulating gene expression that governs cell growth and fibrosis in cardiac muscle, illustrating their potential as therapeutic targets in CVD [10]. These epigenetic modifications play a significant role in the development and progression of CVDs, making them critical targets for potential therapeutic intervention.

CVDs include different disorders of the heart and blood vessels, such as coronary artery disease (CAD), stroke, heart failure, and peripheral artery disease, including atherosclerosis [11]. CVDs are complex disorders influenced by genetic and environmental factors, including gene–environment interactions. Epigenetics is a likely candidate for filling the missing links that lead to cardiovascular pathology [12].

Developing new treatment strategies for heart disease in the era following the mapping of the human genome is critical when considering their potential benefits and disadvantages. Genetics, epigenomic, and environmental influences play a significant role in complex heart diseases such as hypercholesterolemia, valvular diseases, heart failure with preserved/reduced ejection fraction, atherosclerotic heart disease, hypertensive syndromes, primary arrhythmogenic conditions, cardiomyopathies, and congenital heart syndromes [13,14,15]. Moreover, various risk factors are linked to changes in DNA methylation and histone modifications, including abnormal lipid levels, high blood sugar, high blood pressure, excess weight, high levels of homocysteine, aging, pollution, and lifestyle choices such as smoking, poor diet, and lack of physical activity.

The increase in CVDs, such as CAD, stroke, and heart failure, can be attributed to lifestyle factors like sedentary habits and poor diet [16]. In recent decades, CVD outcomes and patient survival rates have improved because of statins, β-blockers, and ACE inhibitors [17]. However, these treatment protocols cannot prevent CVD induction and progression [17]. Thus, the search for new, more efficient CVD treatments is required. Epigenetics, which encompasses modifications such as DNA methylation and histone changes triggered by these lifestyle factors, offers new avenues for therapeutic targets and treatments, potentially reversing the adverse effects of these environmental influences [15]. Given the challenges presented by existing therapies and the complex interplay of genetic and lifestyle factors in CVD, there is a growing interest in exploring natural bioproducts. These substances, particularly those derived from plants, have been recognized for their nutritional benefits and potential to regulate epigenetic mechanisms.

Bioproducts, particularly those derived from plants, have long been recognized for their therapeutic properties, including their role in nutritional interventions to delay the progression of CVDs [18,19,20]. These compounds, rich in bioactive molecules, regulate epigenetic mechanisms such as DNA methylation and histone modifications, potentially treating diseases from atherosclerosis to cancer. Despite extensive research on the effect of numerous dietary and bioactive chemicals, the complexities of how these factors affect epigenetic changes in CVDs are not fully understood. Investigating bioproducts with epigenetic properties (epidrugs) has revealed new therapeutic possibilities for cardiovascular health. These natural compounds from terrestrial and marine ecosystems have shown considerable promise in modulating key CVD-associated epigenetic markers. By highlighting recent breakthroughs, this review emphasizes how these bioproducts, acting as potential epidrugs, may be crucial in revolutionizing CVD management. Integrating these advances into existing medical frameworks may increase therapeutic efficacy against CVDs.

The field of cardiovascular epigenetics has witnessed remarkable evolution over the past two decades, with several breakthrough discoveries transforming our understanding of disease mechanisms and therapeutic approaches. Early investigations (2005–2010) established fundamental connections between DNA methylation patterns and atherosclerosis development, particularly the silencing of protective genes such as KLF2 and KLF4 through promoter hypermethylation [6,21]. The subsequent decade (2010–2018) brought critical insights into histone modifications, with the identification of HDAC9 as a genetic risk factor for atherosclerosis [6] and the recognition of Sirtuin 1 (silent mating type information regulation 2 homolog 1, SIRT1). SIRT1 has cardioprotective effects through the deacetylation of eNOS [22]. Concurrently, the discovery of the role of miR-33a/b in dyslipidemia (2011) [6] and the identification of TET2 deficiency in accelerating atherosclerosis (2017) [23] have highlighted the coordinated involvement of multiple epigenetic mechanisms in cardiovascular disease. Recent advances (2019–2025) have significantly expanded therapeutic possibilities, with the targeting of DOT1L for protection against atherosclerosis via the NF-κB pathway (2022) and the groundbreaking application of base editing techniques for cholesterol management (2023) [24,25]. Most recently, studies have revealed causal relationships between DNA methylation age acceleration and increased risk of cardiac arrhythmias and heart failure (2024), while metabolic–epigenetic crosstalk has emerged as a critical determinant in cardiovascular disease progression (2025) [26]. A 2025 study demonstrated that lactylation, a novel posttranslational modification linking metabolism with epigenetics, plays a crucial role in heart failure pathogenesis by affecting the chromatin structure in cardiomyocytes [27].

Throughout this evolution, natural bioactive compounds have increasingly gained attention for their epigenetic effects on cardiovascular health. Resveratrol, for example, has emerged as a potent activator of SIRT1, preventing endothelial dysfunction by maintaining eNOS activity and reducing cardiac fibrosis through SIRT3-mediated regulation of the TGF-β/SMAD3 pathway [28]. Curcumin demonstrates remarkable cardioprotection by inhibiting p300 histone acetyltransferase (HAT) activity, preventing pathological cardiac hypertrophy, and modulating histone deacetylase 1 (HDAC1) expression to improve vascular structure [29]. Epigallocatechin-3-gallate (EGCG), the primary catechin in green tea, inhibits DNMT1 activity and influences histone acetylation patterns, significantly reducing atherosclerotic lesions and improving cardiac function after pressure overload [30,31]. These natural compounds offer promising therapeutic strategies by targeting key epigenetic mechanisms with potentially fewer side effects than synthetic alternatives, positioning them as valuable agents for cardiovascular disorder prevention and treatment.

This review details how natural bioproducts target epigenetic mechanisms to influence gene expression relevant to cardiovascular health and to prevent and actively manage CVDs. By highlighting innovative approaches and emerging technologies, we aim to provide a comprehensive roadmap for reducing the global impact of CVDs. To address the broad spectrum of CVDs, we predominantly focus on plant-derived bioproducts, such as polyphenols and flavonoids, because of several key factors. First, the extensive body of research available on these compounds provides a robust foundation for discussing their potential in managing and treating CVDs. The mechanisms of action of these bioproducts, especially their roles in epigenetic modulation, are well documented and provide clear pathways through which they influence cardiovascular health. Moreover, several plant-derived compounds are recognized for their safety profiles and therapeutic potential, making them particularly suitable for long-term management strategies for CVDs. The pleiotropic effects of these bioproducts, capable of acting on multiple pathways, offer significant advantages in treating complex diseases involving various physiological processes. While this review emphasizes plant-based bioproducts, it acknowledges the emerging potential of other sources, such as marine-derived bioproducts. The current focus reflects a strategic choice to explore the most substantiated and clinically relevant options in natural therapeutics for cardiovascular health.

## 2. Epigenetics in Cardiovascular Disorders

Epigenetics is the study of heritable changes in gene expression that occur without alterations to the DNA sequence. Epigenetic processes are crucial in gene expression and cellular function. Genetic variation connects with phenotypes through allele-specific genetic modifications. DNA methylation, an important epigenetic mechanism, involves adding methyl groups to the 5’ carbon of cytosine residues within CpG dinucleotides. This process, facilitated by DNA methyltransferases (DNMTs), produces 5-methylcytosine (5mC), altering DNA’s structural integrity and accessibility, thereby impacting gene expression. Typically, DNA methylation acts as a transcriptional repressor by attracting proteins that promote gene silencing, such as methyl-CpG binding proteins [32]. The DNMT family, including DNMT1, DNMT3A, and DNMT3B (with DNMT2 playing a less clear role in this context), is vital for establishing and maintaining these methylation patterns. In addition, the ten-eleven translocation (TET) proteins, TET1, TET2, and TET3, initiate the conversion of 5mC to 5-hydroxymethylcytosine (5hmC), participating in DNA demethylation [33].

Another crucial modification, histone acetylation, involves adding acetyl groups to lysine residues on histone tails, enhancing chromatin decondensation and increasing transcriptional activity. Conversely, HDACs, including the NAD+-dependent sirtuin family (SIRT1–SIRT7), remove these acetyl groups, leading to chromatin condensation and transcriptional repression [34,35]. The SIRT family is a key player in this intricate process with its diverse roles in chromatin remodeling, metabolic control, stress responses, cellular differentiation, the cell cycle, and aging [36,37].

miRNAs are short, non-coding RNAs that control gene expression post-transcriptionally by binding to the 3’ untranslated regions (3’ UTRs) of target mRNAs [38]. This interaction can lead to mRNA degradation or inhibit translation, positioning miRNAs as potential biomarkers and therapeutic targets for cardiovascular and other diseases [38,39]. Recent studies have demonstrated the role of miRNAs in gene regulation, emphasizing their potential for innovative diagnostic and therapeutic applications in personalized medicine, particularly in cancer and CVDs [6,40].

**Figure 1:**
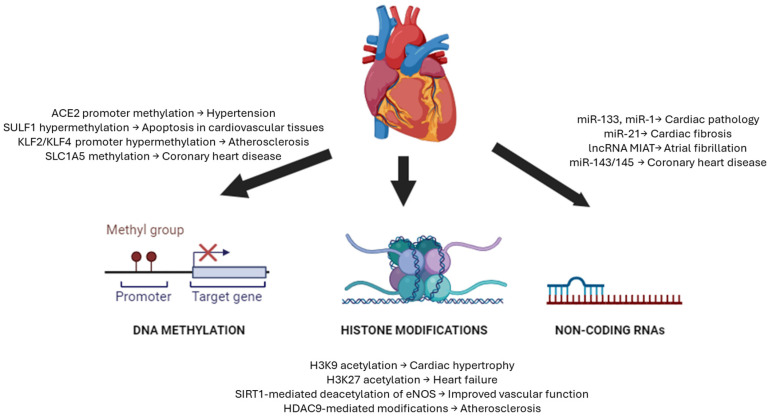
Epigenetic mechanisms in cardiovascular disorders. The heart (**center**) connects to three major epigenetic regulatory mechanisms influencing cardiovascular pathophysiology. DNA methylation (**left**) occurs primarily at CpG islands in gene promoter regions, where DNMTs add methyl groups to cytosine residues, typically repressing gene expression. Specific examples include ACE2 promoter methylation in hypertension, SULF1 hypermethylation in cardiovascular apoptosis, KLF2/KLF4 promoter hypermethylation in atherosclerosis, and SLC1A5 methylation in coronary heart disease. Histone modifications (**center**, **bottom**) involve various chemical changes to histone tails, including H3K9 acetylation in cardiac hypertrophy, H3K27 acetylation in heart failure, SIRT1-mediated deacetylation of eNOS improving vascular function, and HDAC9-mediated modifications in atherosclerosis. Non-coding RNA regulation (**right**) encompasses multiple RNA types that control gene expression without coding for proteins, including miR-133 and miR-1 in cardiac pathology, miR-21 in cardiac fibrosis, lncRNA MIAT in atrial fibrillation, and miR-143/145 in coronary heart disease. These epigenetic mechanisms collectively contribute to various cardiovascular conditions, including atherosclerosis, heart failure, hypertension, coronary heart disease, myocardial infarction, and vascular calcification. DNMTs, DNA methyltransferases; HATs, histone acetyltransferases; HDACs, histone deacetylases; miRNAs, microRNAs; lncRNAs, long non-coding RNAs.

Epigenetics plays a crucial role in controlling the activity and expression of genes linked to CVDs via DNA methylation, histone modifications, and the modulation of non-coding RNAs. These processes strongly influence the progression of CVDs [41] (Figure 1). Understanding these epigenetic processes provides valuable insights into developing novel therapeutic strategies targeting the underlying epigenetic dysregulation in CVDs.

### 2.1. DNA Methylation in Cardiovascular Disorders

DNA methylation plays a vital role in CVDs. The expression of different genes related to coronary heart disease, heart failure, hypertension, and other CVDs is associated with DNA methylation [42] (Table 1). Global methylation is increased in patients with dyslipidemia [43], and there is a positive correlation when comparing global DNA methylation with coronary heart disease [44,45] and acute myocardial infarction [44]. Genes related to cardiovascular processes, such as atherosclerosis, hemostasis, and coagulation, are differentially methylated in CVD [46]. The association of CVD with gene level has been documented in extensive genome-wide association studies (GWAS) [45]. The CG22304262 CpG site affects SLC1A5 expression and correlates with incident coronary heart disease [44]. In acute myocardial infarction mouse models, five genes (*Ptpn6*, *Csf1r*, *Col6a1*, *Cyba*, and *Map3k14*) have been implicated in the acute myocardial infarction pathway by regulating DNA methylation, suggesting their potential as early methylated biomarkers for clinical diagnosis [44].

Numerous studies have explored the correlation between DNA methylation and various stages of atherosclerosis. Four primary pathogenic mechanisms have been proposed: (i) inflammation and endothelial dysfunction, (ii) macrophage and foam cell (FC) formation, (iii) proliferation of vascular smooth muscle cells (VSMCs) and (iv) plaque rupture and thrombosis in atherosclerosis [47]. Regarding the impact of abnormal DNA methylation on inflammation and endothelial dysfunction in atherosclerosis, key protective factors such as KLF2, KLF4, and CREG exhibit anti-atherosclerotic actions. KLFs, a subset of zinc finger transcription factors, regulate cell differentiation and proliferation. KLF2, an anti-inflammatory transcription factor, controls adipocyte differentiation and is downregulated in human umbilical vein endothelial cells (HUVECs) treated with oxidized low-density lipoprotein (LDL) through DNMT1-mediated promoter methylation [48]. Similarly, the overexpression of *DNMT3a* and *DNMT3b* causes hypermethylation of the *CREG* and *KLF4* promoters, suppressing their expression and promoting endothelial dysfunction [49]. DNMT inhibitors such as RG108, N-acetylcysteine, and 5-aza-2′-deoxycytidine restore hypermethylation in endothelial cells, but their clinical applications for treating atherosclerosis are pending U.S. Food and Drug Administration (FDA) approval [50]. Macrophages engulf ox-LDL and form lipid streaks in atherosclerosis, influenced by abnormal DNA methylation. Homocysteine independently increases atherosclerosis risk by inducing lipid accumulation. DNMT3b increases homocysteine-induced atherosclerosis by inhibiting *SCARB1* expression. DNMT1-regulated DNA methylation targets Peroxisome proliferator-activated receptor γ (PPAR-γ), and 5-aza-2′-deoxycytidine (5-Aza-Dc) may counteract infiltration and activate immune cells, exerting protective effects against atherosclerosis.

Abnormal DNA methylation in vascular smooth muscle cells (VSMCs) plays a significant role in atherosclerosis. Patients with advanced atherosclerosis exhibit genome hypomethylation in CpG islands of atherosclerotic plaques [51]. Homocysteine increases PDGF methylation by inhibiting DNMT1, which leads to VSMC proliferation and migration [52].

TET methylcytosine dioxygenase 2 (TET2) mediates DNA demethylation by converting 5-methylcytosine to 5-hydroxymethylcytosine, affecting VSMC phenotype, endothelial dysfunction, and macrophage inflammation [53]. TET2, through its role in DNA demethylation, potentially influences the expression of important vascular smooth muscle cell genes such as myocardin (*MYOCD*) and serum response factor (*SRF*), which are crucial for smooth muscle function and differentiation in atherosclerosis. In addition, TET2 may affect the regulation of *KLF4*, a gene involved in phenotypic switching of vascular smooth muscle cells, a process central to the pathology of atherosclerosis [47]. Furthermore, decreased *DNMT3b*/*DNMT1* expression in smooth muscle cells increases *MMP2*, *MMP9*, and *PDGF* expression.

**Table 1:** Regulation of DNA methylation in CVDs.

Diseases	Target	Mechanism	Reference
Coronary heart disease	cg22304262	Alters *SLC1A5* amino acid transporter expression	[44]
	Global DNA	Hypermethylation correlates with increased disease risk	[44,45]
	cg04988978	Modulates *MPO* expression affecting vascular inflammation	[54]
Acute myocardial infarction (MI)	Global DNA	Differential methylation of genes involved in MI pathways	[44]
Heart failure	DNMT3a	Impairs cardiomyocyte metabolism and contractility	[55]
	CTGF, MMP2, miRNA-155, HEY2, MSR1, MYOM3, COX17, miRNA-24-1	Alters methylation patterns affecting cardiac remodeling	[56]
	KCNA4, KCNIP4, SMOC2	Regulates cardiac ion channel function	[57]
	DNMT2, glutathione peroxidase 1	Mediates oxidative stress response in cardiomyocytes	[58]
Vascular calcification	DNMT3b, H19	Promotes osteogenic transdifferentiation of VSMCs	[59]
	G3BP1	Mediates Wnt signaling in arterial calcification	[60]
	SM22a	Regulates VSMC phenotypic switching during calcification	[61]
Hypertension	mitochondrial fusion 2	Regulates mitochondrial dynamics affecting vascular function	[62]
	Interferon	Modulates immune response in essential hypertension	[63]

### 2.2. Histone Modifications in Cardiovascular Disorders

Abnormal histone modification can disrupt the balance of gene expression linked to CVDs, leading to alterations in cellular phenotypes and cardiac function [6]. Key histone methylation and acetylation processes critically influence the onset and progression of CVDs by modulating pathophysiological pathways within the cardiovascular system (Table 2). Histone methylation, particularly on lysine and arginine residues of histones, is a critical regulatory mechanism in gene expression. Specific enzymes, such as histone methyltransferases (HMTs) and histone demethylases (HDMs), add or remove methyl groups, respectively. Depending on the specific histone tail and modified residue, this modulation can either activate or repress gene expression. For example, methylation of histone H3 on lysine 4 (H3K4) is generally associated with transcriptional activation, whereas methylation on H3K9 is associated with repression [64]. Histone acetylation, mediated by HATs and HDACs, plays a similarly crucial role in CVDs. The acetylation of histones generally results in an open chromatin structure that promotes gene transcription. In cardiovascular contexts, changes in acetylation levels are linked to conditions such as cardiac hypertrophy and heart failure. For example, acetylation at H3K9 and H3K27 has been detected in cardiac hypertrophy and heart failure, suggesting a direct impact on gene expression [65]. The interaction between histone modifications and other epigenetic mechanisms, such as DNA methylation and non-coding RNAs such as miRNAs, create a complex regulatory network. These interactions may influence cardiovascular health by modulating the expression of genes critical for heart function and vascular integrity [64,65].

### 2.3. Non-Coding RNAs in Cardiovascular Disorders

Non-coding RNAs exert substantial regulatory control over the pathophysiological and physiological mechanisms underlying CVDs, including coronary heart disease, myocardial infarction, and vascular calcification (Table 3). These non-coding RNAs demonstrate cell and organ specificity in their expression patterns. They are detectable in human blood, urine, and other bodily fluids. Given their high sensitivity, stability, and ease of detection, non-coding RNAs demonstrate significant potential as novel biomarkers for the diagnosis and prognosis of CVDs [6].

## 3. Treatment of Cardiovascular Disorders with Epibioproducts

Epigenetic regulation plays an essential role in modulating the expression of genes associated with various aspects of CVD therapy, including pathogenesis, mechanistic pathways, metabolic processes, transporter functions, and pleiotropic effects [6,115]. Pharmacogenetic studies have shown the significance of epigenetic mechanisms in determining the efficacy and safety of conventional medications used to manage CVD [26]. Many epigenetic changes are reversible, sparking interest in bioproducts, particularly those with documented epigenetic effects, and emerging epigenetic drugs known for their minimal side effects and ability to penetrate cardiac tissues [116]. These interventions show potential for preventing and treating CVD, especially in its early stages [26]. The increasing number of clinical trials examining the therapeutic potential of such epigenetic drugs reflects their growing importance. This class of pharmacological agents, designed to specifically target the cardiac epigenome or enzymes involved in epigenetic modulation, is known as “epigenetic drugs.” Naturally occurring compounds can modulate DNA methylation, chromatin structure, and miRNA expression, thus mediating dietary effects on epigenetic mechanisms related to CVD.

### 3.1. Natural DNA Methylation Modifiers

#### 3.1.1. Polyphenols and Flavonoids

Polyphenols and flavonoids are widely distributed in vegetables, fruits, green tea, red wine, cocoa, and other food components and can affect epigenetic pathways [18]. There are a variety of biologically active polyphenols, such as resveratrol, catechin, epicatechin, and epigallocatechin-3-o gallate. Natural polyphenols and flavonoids exert cardioprotective effects through pleiotropic mechanisms, including anti-inflammatory, antioxidant, anti-thrombotic, and vasodilatory activity, improving endothelial function [18].

Polyphenols and flavonoids also affect DNA methylation by suppressing DNMT activity [30]. Certain drugs already available, including cardiovascular medications procaine, procainamide, and hydralazine, suppress DNMT activity and may also influence unidentified epigenetic mechanisms [117]. Nonetheless, the adverse effects associated with these drugs pose significant concerns. Natural epigenetic bioproducts could help mitigate the harmful effects of other drugs. For example, pairing the prospective anti-cancer drug dichloroacetate (DCA), which alters cancer cell metabolism, with a compound that blocks the methylation of the SLC5A8 transporter may lower the required doses of DCA [118]. This approach aims to similarly reduce adverse side effects while maintaining the efficacy of the drugs against CVD drugs and their effectiveness against cancer.

Catechin, epicatechin, and EGCG are natural antioxidants in green tea. Catechin and epicatechin increase the formation of S-adenosyl-L-homocysteine (SAH), a potent noncompetitive inhibitor of DNMTs, via inhibiting catechol-O-methyltransferase (COMT)-mediated O-methylation of catechols and catecholamines [119]. In addition, 3-O-(3,4,5-trimethoxybenzoyl)-epicatechin inhibits methylenetetrahydrofolate (MTHFR) activity, blocking the folate cycle necessary for the conversion of homocysteine to methionine, a precursor of SAM that serves as a methyl donor in DNA methylation [120]. EGCG is the most abundant catechin and primary polyphenol in green tea. It differs from epicatechin primarily due to the addition of a gallate group, which significantly increases its antioxidant properties [121]. EGCG has many potential health benefits, including neuroprotective effects, cancer prevention properties, and the ability to improve cardiac health. EGCG is the most potent DNMT1 inhibitor and inhibits catalytic activity through the gallic acid moiety [30]. EGCG also can inhibit in vitro activity of DNMT3a and DNMT3b [122]. The epigenetic potential of EGCG extends beyond DNA methylation and includes the modulation of chromatin architecture through histone post-translational modifications. EGCG influences the expression of enzymes involved in acetylation and methylation, thereby impacting heterochromatin formation. Its effects are evident in altering chromatin architecture through histone protein acetylation and methylation, and DNA methylation patterns. This modulation occurs through direct effects on histone marks and indirectly via the inhibition of DNMT activity by EGCG, reactivating genes silenced by methylation [31,123].

EGCG reduces atherosclerotic lesions and SREBP1 and increases LXRa and LXRP [1]. A meta-analysis to evaluate the effectiveness of EGCG in mitigating myocardial ischemia–reperfusion injury showed that EGCG is cardioprotective by substantially reducing myocardial infarction size compared to the control groups; EGCG exhibits marked improvements in heart function, serum myocardial injury enzyme levels, and oxidative stress levels in animal models of MIRI. Liu et al. (2018) [53] found that EGCG therapy significantly reversed the decreased expression of SERCA2a, a critical protein, after cardiac pressure overload in mice. They observed an increase in acetylated histone H3 and H3K9 levels and a decrease in the activity and binding of HDAC1 after EGCG therapy. Those findings showed that EGCG inhibits HDAC1, allowing acetylated histones to bind to the promoter of the *SERCA2a* gene, thereby increasing its expression. These observations suggest that histone acetylation plays a role in the ability of EGCG to prevent heart failure caused by pressure overload. Despite these effects, the specific epigenetic mechanism through which EGCG prevents heart failure remains elusive.

Resveratrol, a polyphenol compound found in vegetables, peanuts, berries, and grapes, has anti-inflammatory and antioxidant properties [8]. Resveratrol exerts cardioprotective activities by modulating the expression of genes related to inflammation, redox enzymes, eNOS, signaling kinases, adhesion molecules, immune cell function, and vascular remodeling [124]. Excess levels of ROS and nitrogen species, such as superoxide (O_2_−), hydroxyl radical (OH), hydrogen peroxide (H_2_O_2_), and peroxynitrite, are directly scavenged by resveratrol, preventing lipid peroxidation and DNA damage [125,126]. Resveratrol also increases the quantity of antioxidants and protects endothelial cells and cardiomyocytes from oxidative damage [127]. Resveratrol further reduces ROS generation by inhibiting various isoforms of NADPH oxidases and altering the activity of mitochondrial respiratory chain enzymes [128]. It accomplishes this by reducing the activity of NOX isoforms via SIRT1 upregulation, which then deacetylates NF-κB [128]. Resveratrol treats deoxycorticosterone acetate (DOCA) salt hypertension by methylating the vascular H3K27me3 mark [129] and improving coronary heart disease, atherosclerosis, and metabolic disorders through increased SIRT1 expression in endothelial cells [28]. Resveratrol and its derivatives act as DNMT3 inhibitors [124]. Resveratrol suppresses the proliferation of smooth muscle cells by blocking the hypermethylation of phosphatase and tensin homolog (PTEN) induced by homocysteine; this effect is associated with reduced DNMT1 expression [130].

Long-term resveratrol use activates SIRT1, reduces FOXO1-related pro-apoptotic signaling, increases PCC-1 α and mitochondrial biogenesis, improves myocardial function, and prevents Ang-II-induced cardiac remodeling in aging mice [131,132]. Moreover, resveratrol reduces IL-6 activation mediated by SIRT1, protects against Ang-II-induced hypertrophy in H9c2 cells [133], and aids in vascular remodeling while preventing pulmonary hypertension development.

Quercetin, puerarin, and other natural flavonoids exert antioxidant and anti-inflammatory effects through changes in gene expression. Quercetin decreases the total activity of DNMTs and the DNA methylation level at the NF-E2-related factor 2 (*Nrf2*) gene promoter [134], which plays a significant role in antioxidant signaling pathways crucial for cardiovascular health [135]. Puerarin, derived from the plant *Pueraria lobata*, exhibits various cardiovascular benefits. It has positive effects against CBDs through its anti-inflammatory, antioxidant, and vasodilatory properties. The influence of Puerarin on CVDs extends to modifying molecular pathways that could potentially involve epigenetic mechanisms; however, direct evidence of its effects on DNA methylation in cardiovascular contexts is sparse [136].

The role of curcumin as a DNMT1 inhibitor is under debate. Some studies, such as those by Liu et al., have suggested that curcumin can covalently bind to the catalytic thiolate of Cys 1226 of DNMT1 and induce global DNA hypomethylation [137]. In contrast, others did not confirm the DNA methylation inhibitory activity of curcumin [135,138].

#### 3.1.2. Folic Acid and Other B Vitamins as Methyl Donors

Although dietary compounds are generally deemed safe, the intricate chemical structures of many natural substances often result in limited data regarding their toxicity. Consequently, assuming complete biological safety is unwarranted. Specific food components may confer benefits at normal physiological levels but pose risks at higher, pharmaceutical-like doses. Following ingestion, folate (vitamin B9) is converted to tetrahydrofolate, which is crucial for converting homocysteine to methionine [139]. Similarly, S-adenosylmethionine (SAM) is converted into S-adenosyl-L-homocysteine (SAH) and acts as a methylation inhibitor. Folate and other B vitamins (B6 and B12) are involved in one-carbon metabolism with methionine, which is essential for SAM production and serves as a methyl group donor [140]. Folate deficiency is associated with genome-wide DNA hypomethylation and correlates with higher CVD risk [141]. Folate treatment increases global DNA methylation levels [142]. Indeed, decreased levels of folic acid correlate with higher serum levels of homocysteine, a metabolic risk of CVD [143].

Dietary supplementation with folic acid is expected to decrease CVD risk. However, most large-scale clinical interventions failed to show the beneficial effects of dietary folate on CVD and coronary heart disease, with modest positive effects on stroke prevention [144].

#### 3.1.3. RCI-1502

LipoEsar is a bioproduct and lipoprotein complex derived from the dorsal muscle of the European *Sardina pilchardus* Walbaum, 1792 [145] and is the structural base for RCI-1502. LipoEsar retains the natural properties of its active components. LipoEsar is rich in proteins and fatty acids, with palmitic acid as the primary saturated fatty acid and oleic and palmitoleic acids as the primary monounsaturated fatty acids. Moreover, it contains eicosapentaenoic acid (EPA), eicosatetraenoic acid (ETA), and docosahexaenoic acid (DHA) as the primary polyunsaturated fatty acids. It is also abundant in potassium, phosphorus, calcium, and vitamins B5 and C [145]. Preclinically, LipoEsar (i) lowers serum cholesterol levels, (ii) decreases serum triglyceride levels, (iii) reduces serum glucose levels, (iv) regulates various immunological factors, including increasing white blood cell counts, lymphocytes, and monocytes, and (v) reduces the presence of ameboid microglial cells [145,146]. LipoEsar, administered at a dosage of 750 mg/day for three months, mitigates cardiovascular risk factors in healthy individuals and patients with chronic hyperlipidemia [145]. Clinical trials reveal that LipoEsar effectively reduces total cholesterol, low-density lipoprotein (LDL) cholesterol, blood glucose, and uric acid levels while simultaneously elevating high-density lipoprotein (HDL) cholesterol levels. In individuals with chronic hyperlipidemia, LipoEsar (1500 mg/day/three months) decreases the size of xanthelasma plaques, which are indicators of high blood cholesterol levels, and reduces the size of atherosclerotic plaques on the aortic wall [147].

Omega-3 fatty acids, specifically EPA and DHA, effectively reduce triglyceride levels and are recommended for coronary heart disease and hypertriglyceridemia [148]. A diet high in PUFAs is beneficial to maintaining healthy blood cholesterol levels, which can significantly reduce cardiovascular risk [149]. These fatty acids have antiarrhythmic effects, preventing calcium overload in cardiac myocytes under stress, and contribute to cell membrane protein modulation and gene expression [150]. Omega-3 fatty acids also increase the secretion of anti-inflammatory prostaglandins, contrasting with omega-6 fatty acids, which generate proinflammatory mediators [151]. Moreover, omega-3 polyunsaturated fatty acids (PUFAs) help manage dyslipidemia. They also lower systolic and diastolic blood pressures, improve endothelial function, and reduce the risk of sudden cardiac death [152,153].

RCI-1502 has a different fatty acid profile and a higher protein content than LipoEsar. RCI-1502 also includes vital vitamins B5 and C and essential minerals such as potassium, phosphorus, and calcium. Our group recently examined the therapeutic potential of RCI-1502 on gene expression and DNA methylation in HFD-fed mice and patients with dyslipidemia. LC-MS/MS showed that 75 proteins in RCI-1502 are predominantly engaged in binding and catalytic activity and regulate pathways associated with CVD. In HFD-fed mice, RCI-1502 therapy reduced the expression of CVD-related genes such as vascular cell adhesion molecule and angiotensin. RCI-1502 also reduced DNA methylation levels, higher in HFD-fed mice, to levels comparable to control animals. Moreover, dyslipidemic patients exhibited higher DNA methylation levels in peripheral blood leukocyte DNA compared to healthy subjects, suggesting a potential association with cardiovascular risk. These individuals received RCI-1502 orally over four weeks. A significant (*p* < 0.05) decrease in DNA methylation levels was observed only among patients with dyslipidemia, while no significant change occurred in healthy subjects.

Serum analysis further revealed that RCI-1502 effectively reduced cholesterol and triglyceride levels in patients with dyslipidemia. These findings suggest that RCI-1502 may serve as an epigenetic modulator for treating cardiovascular disorders, particularly dyslipidemia. Proteomic investigation of RCI-1502 revealed the presence of fumarate, known to inhibit α-ketoglutarate-dependent dioxygenases responsible for DNA and histone demethylation [154]. RCI-1502 is rich in omega-3 fatty acids [155], and supplementation with omega-3 reduces DNA methylation in blood leukocytes [156].

Indeed, RCI-1502 treatment reduces hypercholesterolemia and hypertriglyceridemia [41]. RCI-1502 is also an epigenetic regulator that alters the expression of cardiovascular-related genes such as *APOE*, *VCAM*, *AGT*, *ACE*, and *ABCB7* [41]. RCI-1502 treatment restored *VCAM* expression to baseline and *AGT*- and *ACE* mRNA levels, which the HFD had significantly increased. Furthermore, RCI-1502 therapy increased *ABCB7* mRNA expression, which is related to the deterioration of cardiac function caused by chronic pressure overload. These findings point to the potential therapeutic applicability of RCI-1502 in managing CVDs via modulating key gene expression pathways.

This section discussed the potential of various natural bioproducts, such as polyphenols, flavonoids, and other plant-derived compounds, in modulating DNA methylation processes relevant to cardiovascular health (Table 4). These findings pave the way for novel epigenetic therapies leveraging the DNA methylation-modifying properties of natural compounds.

### 3.2. Natural Compounds Regulating Histone Modifications

#### Natural HDAC and HAT Modifiers

**Figure 2:**
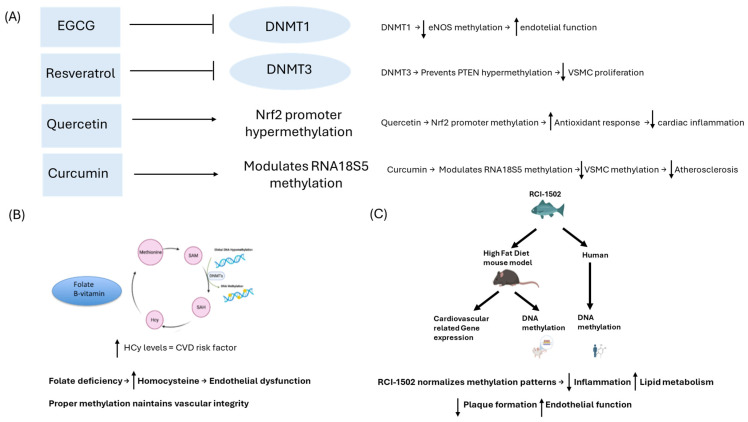
Schematic representation of how natural bioproducts influence DNA methylation processes with implications for cardiovascular health. (**A**) Natural compounds (EGCG, resveratrol, quercetin, and curcumin) modulate DNA methylation pathways to regulate gene expression linked to cardiovascular outcomes. EGCG inhibits DNMT1, reducing eNOS promoter methylation and increasing endothelial function. Resveratrol inhibits DNMT3, preventing PTEN hypermethylation and decreasing VSMC proliferation. Quercetin decreases *Nrf2* promoter methylation, increasing antioxidant responses and reducing cardiac inflammation. Curcumin modulates RNA18S5 methylation, attenuating VSMC proliferation and atherosclerosis. (**B**) Folate, a B vitamin, is crucial in the methionine cycle and cardiovascular health. Folate converts homocysteine (Hcy) to methionine, forming S-adenosylmethionine (SAM), the cell’s primary methyl donor. Folate deficiency leads to hyperhomocysteinemia, a risk factor for endothelial dysfunction and atherosclerosis. (**C**) RCI-1502, a novel epibioproduct sourced from the dorsal muscle of *S. pilchardus* Walbaum, normalizes DNA methylation patterns of these genes, reducing inflammation, improving lipid metabolism, and decreasing atherosclerotic plaque formation. EGCG, epigallocatechin gallate; eNOS, Endothelial nitric oxide synthase; Hcy, homocysteine; HFD, high-fat diet; SAM, S-adenosylmethionine; SAH, S-adenosylhomocysteine.

The polyphenol curcumin, the yellow pigment found in turmeric rhizomes (*Curcuma longa*), exhibits promising therapeutic potential by influencing epigenetic mechanisms (Figure 2). It is the most common HAT inhibitor (HATi) studied in heart disease. Turmeric is generally regarded as safe by the US Food and Drug Administration, with minimal toxicity [157], and is non-genotoxic and non-mutagenic [30,158]. Furthermore, the administration of curcumin (at dosages ranging from 500 to 8000 mg/day for three months) to individuals with cardiovascular risk factors is associated with a favorable safety profile [159].

The benefits of curcumin extend across various forms of cardiovascular pathologies, including atherosclerosis, heart failure, aortic aneurysm, stroke, myocardial infarction, and complications arising from diabetes [160,161]. Curcumin attenuates endothelial dysfunction in stroke; the antioxidant-mediated inhibition of LDL oxidation contributes to atherosclerosis and preserves cardiac function by preventing cardiomyocyte apoptosis and subsequent fibrotic remodeling post-myocardial infarction.

The p300/CREB-binding proteins (CBPs) are a ubiquitous family of HATs serving as transcriptional coactivators, crucial for cellular function and survival. Curcumin induces proteasome-dependent protein degradation of p300 and inhibits its acetyltransferase activity, affecting substrates such as H3 or p53 [162]. Radiolabeled curcumin demonstrates covalent association with p300, inhibiting its HAT activity. Histone acetylation regulates genes involved in myocardial hypertrophy, an adaptive response to stresses such as increased afterload or myocardial infarction [65]. p300-HAT is a coactivator for transcription factors implicated in cardiac hypertrophy, influencing gene expression [163]. Increased transcriptional activity of p300 is observed in agonist-induced cardiac hypertrophy and is reversible by inhibiting p300-HAT activity [164].

Curcumin treatment in hypertensive rats restores systolic function and downregulates the hypertrophic transcription factor GATA4 [165]. In rats with myocardial infarction, curcumin improves systolic function and prevents hypertrophy. It also inhibits the formation of the p300/GATA4 complex and suppresses hypertrophic responses in rat cardiomyocytes stimulated by agonists or p300.

Curcumin activates Nrf2, leading to the induction of HO-1, which confers cytoprotective and anti-inflammatory responses against oxidative stress [166]. Moreover, curcumin’s inhibition of p300 HAT and the subsequent downregulation of NF-κB improve endothelial function and reduce inflammatory monocyte adhesion [167,168]. Another significant aspect of curcumin’s action is the downregulation of AT1R, thus interfering with angiotensin II signaling and its detrimental cardiovascular effects [169]. Furthermore, curcumin modulates key signaling molecules such as p38 MAPK, JNK, and ASK1, potentially attenuating chronic heart failure [170]. By targeting these diverse molecular pathways, curcumin presents a promising natural adjunct in managing and preventing CVDs.

Curcumin is a broad-spectrum epigenetic modulator that influences the activity of DNMT1, HATs, and HDACs [171]. Curcumin has a potent HDAC inhibitory effect that exceeds the inhibitory capacity of well-known HDAC inhibitors such as sodium butyrate and valproate [172]. Curcumin reduces the activity of HDAC1, 3, and 8 [172]. In contrast, curcumin activates HDAC2, increasing the activity and expression of this deacetylase [173].

Curcumin treatment reduces *HDAC1*, *MMP-2*, and *TGFβ* expression. Curcumin induces *TIMP1* transcriptional activation in hypertensive rats by suppressing *HDAC1* expression while increasing histone H3 acetylation at the *TIMP1* promoter region. Furthermore, curcumin reduces extracellular matrix breakdown and interstitial fibrosis caused by hypertension, thus lowering blood pressure. Curcumin may improve vascular structure by reducing *HDAC1* expression, activating TIMP1, and suppressing MMP-2 and TGFβ [174].

HDAC2 is vasculoprotective by preventing vessel inflammation and pathogenic remodeling, as observed in atherosclerosis and other CVDs [175]. Curcumin exerts cytoprotective effects on endothelial function by preventing platelet adhesion to endothelial cells [176] and delaying oxidative stress-induced premature cell senescence by activating deacetylase SIRT1 [177]. Curcumin modulates the methylation patterns at the *RNA18S5* promoter site, reducing rDNA transcription and contributing to the inhibition of VSMC proliferation; this consequently slows the progression of atherosclerosis. In addition, molecular docking analyses suggest that curcumin interacts directly with DNMT1, inhibiting enzyme activity.

**Figure 3:**
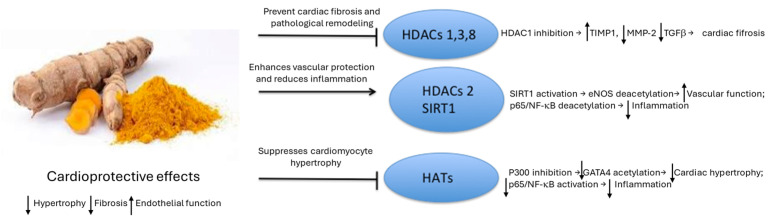
The modulatory effects of curcumin on HDACs and HATs in cardiovascular disease. This schematic shows the molecular interaction between curcumin (**left**), an active component of turmeric, and key enzymes (**right**) involved in regulating histone acetylation. Curcumin inhibits HDAC1, HDAC3, and HDAC8 (class I HDACs), preventing cardiac fibrosis by increasing TIMP1 expression while reducing MMP-2 and TGFβ. Curcumin activates HDAC2 and SIRT1 (a class III sirtuin HDAC), with SIRT1 activation leading to te deacetylation of eNOS (increasing vascular function) and p65/NF-κB (reducing inflammation). Curcumin also inhibits HATs such as p300, preventing GATA4 acetylation and cardiac hypertrophy. These epigenetic modifications lead to reduced cardiac hypertrophy, decreased fibrosis, improved endothelial function, and attenuated progression of atherosclerosis. HATs, histone acetyltransferases; HDACs, histone deacetylases.

Curcumin-dependent activation of SIRT1 also leads to cardioprotective effects by inhibiting apoptosis via the induction of anti-apoptotic Bcl-2 and downregulating multiple proapoptotic factors such as Bax, beclin-1, and BNIP3 in cardiomyocytes [178], suppressing profibrotic activity in cardiac fibroblasts [179], and reducing oxidative stress-induced mitochondrial damage in heart muscle cells [180] (Figure 3).

Curcumin downregulates HATs such as p300/CBP [180] and Gcn5-related N-acetyltransferase (GNAT)-mediated acetylation of histone 3 [181]. As an antioxidant, curcumin can influence acetylation/deacetylation by regulating oxidative stress [182] (Figure 3).

Resveratrol-induced activation of SIRT1 plays a pivotal role in mediating its various gene expression activities. SIRT1 directly preserves the NO-producing function of eNOS by inhibiting oxidative stress-induced acetylation, thus maintaining enzyme activity [28,183]. Moreover, resveratrol mitigates cardiac fibrosis by activating SIRT3 and modulating the TGFβ/SMAD3 pathway in fibroblasts, cardiac myofibroblasts, and cardiomyocytes [184]. This downregulation of tissue fibrosis is further facilitated by SIRT1’s influence on TGFβ/SMAD3-dependent fibrogenesis [185].

### 3.3. Natural Non-Coding RNA Modifiers

Several natural compounds affect the expression of various microRNAs (Table 5), regulating essential cellular functions such as inhibiting cell growth, reducing inflammation, and promoting cell differentiation and cell cycle arrest [186].

#### 3.3.1. Natural miRNAs Modifiers

Under tumor necrosis factor α (TNFα)-induced inflammatory conditions, resveratrol attenuates endothelial inflammation and induces SIRT1 expression in endothelial cells. The expression of miR221/222 is suppressed in TNFα-treated endothelial cells, which is reversed by resveratrol treatment [187]. Resveratrol inhibits endothelial cell apoptosis by upregulating miR-126 expression [188]. A low dose of resveratrol increases re-endothelization ex vivo and reduces neointima formation in vivo after endothelial injury [189]. This role of resveratrol is realized by inhibiting miR-21 expression in endothelial stem/progenitor cells, leading to endothelial formation [190]. Resveratrol treatment causes considerable downregulation of miR-21 expression and cardioprotection against ischemia/reperfusion injury [191].

The suppressive effect of resveratrol on miR-34a expression upregulation plays an important role in SIRT1 restoration in cardiomyocytes after cardiac injury inflicted by ischemia–reperfusion [192]. Resveratrol also reduced miR-34a expression in a rat myocardial injury model [193] and myocardial fibrosis in an in vitro model with TGFβ [194]. The administration of resveratrol decreases the expression of miR-155 in a dose-dependent manner in vitro and attenuates cardiac myocyte hypertrophy in vivo and in vitro [195]. Resveratrol impairs lipopolysaccharide-induced upregulation of miR-155 expression in human peripheral blood monocytes [196], macrophages [197], ischemic brain [198], and adipocytes [139], suggesting that resveratrol modulates miR-155 expression and reduces the inflammatory response and confers protection against diabetes, atherosclerosis, and hypertension [139]. One year of supplementation of resveratrol-enriched grape extract in hypertensive patients with artery coronary disease reduced proinflammatory cytokines via regulation of the expression levels of miR-21, miR-155, and miR-34a [199].

Like resveratrol, other natural compounds, including flavonoids such as kaempferol and quercetin, have been studied for their role in modulating miRNA expression linked to cardiovascular health. The most well-known and researched flavonoids are kaempferol and quercetin. Kaempferol treatment can improve cardiac function and suppress myocardial infarction by decreasing myocardial infarct size, cardiomyocyte apoptosis, oxidative stress, and inflammatory response [200]. Kaempferol inhibits VSCM proliferation and migration by inducing miR-21 expression, thereby showing preventive effects against CVDs [201]. Enhancing miR-21 expression also mediates kaempferol-induced cardiomyocyte protection against myocardial injury by reducing oxidative stress [202]. Quercetin is the most common and widely distributed flavonol compound, and it is present in tea, onion, lettuce, broccoli, beans, and buckwheat [203]. The anti-inflammatory properties of quercetin include the downregulation of miR-155 and the enhancement of miR-21 expression, which is a proinflammatory miRNA [204]. 

Beyond these flavonoids, genistein and crocin also demonstrate protective effects against oxidative stress and inflammation through miRNA modulation. Genistein, another isoflavonoid present in soy, decreases the expression of miR-34a and miR-155 in endothelial cells under oxidative damage and inflammation [205]. Crocin is a carotenoid with antioxidant effects [206] that exerts cardioprotective effects against neuroinflammation and oxidative stress [207]. miR-34a expression is altered in the myocardium after myocardial injury, and the overexpression of miR-34a aggravates this injury by increasing infarct size and decreasing LV function [208]. Crocin exerts cardioprotective effects against myocardial injury by suppressing miR-34a upregulation and enhancing *SIRT1* expression [209]. In contrast, crocin treatment also increases miR-126 and miR-210 expression in rat cardiac tissue, and increased miR-126 and miR-210 expression induces capillary formation and improves cardiac angiogenesis [210]. Pterostilbene, a resveratrol analog, reduces cardiac ROS production and alleviates myocardial injury by increasing miR-15b expression in cardiomyocytes [211].

*Nelumbo nucifera* leaf polyphenol extract and its main component, gallic acid, inhibited VSMC proliferation and migration to decelerate atherosclerosis progression by downregulating miR-21 expression and upregulating miR-145 expression [212]. Gallic treatment increased miR-126 and miR-210 expression in rat myocardial tissue, increased cardiac angiogenesis, and improved serum lipid profile [213]. 

Apigenin, a major plant flavone present in chamomile, can alleviate myocardial infarct and acute myocardial infarct [214]. Apigenin plays a role in conferring protection against myocardial injury by reducing miR-15b expression [214]. Apigenin also attenuates TGF-β-stimulated cardiac fibroblast differentiation, and its mechanisms are associated with miR-155-5p expression [215]. Luteolin is another major flavonoid, and it is present in thyme, carrots, artichokes, basil, cauliflower, celery, and parsley and possesses antioxidant activities and cardioprotective properties [216]. Luteolin increases cell viability dose-dependently and reduces lactate dehydrogenase (LDH) release, thus lowering cardiomyocyte damage and preventing necrosis [217]. Luteolin also mitigated ischemic reperfusion injury and cardiomyocyte apoptosis by inhibiting caspase-3, Bcl-2, and Bax activities, as well as cytochrome C, a significant contributor to myocardial infarctions [217].

Luteolin suppresses oxidative stress and cardiac fibrosis, and its effect is strongly associated with the reduction in miR-21 expression in an in vivo mouse model of myocardial injury [216]. Ampelopsin is a flavonoid with anti-inflammatory, antioxidant, and anti-tumor properties [218]. Ampelopsin decreases miR-21 expression, improves endothelial function, and inhibits vascular inflammation and plaque formation in in vivo models of atherosclerosis. Puerarin, an isoflavone obtained from the root of the kudzu plant, has remarkable activities in treating CVDs [219]. Puerarin increases endogenous miR-21 expression, significantly exerting cardioprotective effects in an in vitro myocardial injury model [219]. Puerarin attenuates cardiac hypertrophy by promoting miR-15b expression in a mouse model of cardiac hypertrophy and primary cardiomyocytes [220].

Geniposide is an iridoid compound extracted from gardenia fruit that is cardioprotective. Geniposide treatment reduces plaque size and alleviates atherosclerosis-associated inflammatory injury. Geniposide treatment reduces plaque size and alleviates atherosclerosis-associated inflammatory injury [221]. Geniposide attenuates endothelial injury and ROS generation by decreasing antioxidant enzyme activities, which are directly associated with the increase in miR-21 expression in animal models of atherosclerosis [222]. 

#### 3.3.2. Natural LncRNAs Modifiers

Calycosin, genistein, and corylin are isoflavones in various plants, including traditional Chinese herbs such as *Radix Astragali* and *Psoralea corylifolia*. Calycosin, an O-mediated isoflavone, has been widely used to alleviate symptoms of diabetes and diabetic nephropathy [11]. Increasing evidence from in vitro and in vivo studies indicates the crucial roles of long non-coding RNAs (lncRNAs) in mediating the physiological effects of calycosin, particularly in breast cancer [11]. EGCG downregulates 3-hydroxy-3-methylglutaryl coenzyme A reductase (*HMGCR*) mRNA expression and upregulates lncRNA *AT102202* expression, highlighting the importance of lncRNAs in cholesterol metabolism [223] and CVDs [224].

Anthoxanthins, a class of plant pigments that include flavonoids such as flavones (e.g., luteolin) and flavonols (e.g., quercetin), are known for their antioxidant properties and are associated with a reduced risk of developing CVDs. Many of these flavonoids, including quercetin, exert their beneficial effects through interactions with lncRNAs. For example, quercetin is linked to the MALAT1/PI3K/AKT signaling pathway, suggesting its potential role in regulating myocardial disease [225].

Ten lncRNAs that influence CVDs and are involved in the flavonoid intervention process include *NKILA*, *BANCR*, *AK021954*, *MEG3*, *HOTAIR*, *GAS5*, *NEAT1*, *AT102202*, *MALAT1*, and *ATB* [11].

Clinical trial information was retrieved from ClinicalTrials.gov, a database maintained by the U.S. National Library of Medicine. Trial identifiers (NCT numbers) are included for reference.

### 3.4. Conclusions

CVDs, including CAD, stroke, heart failure, and peripheral artery disease, are the leading cause of morbidity and mortality worldwide, affecting populations in developed and developing countries. These conditions are complex, arising from genetic, epigenetic, and environmental factors, with lifestyle choices and diet playing significant roles. Despite the complexities involved, our understanding of the epigenetics behind vascular diseases and significant risk factors such as hypertension remains limited.

While natural bioproducts show promise for epigenetic modulation in CVDs, there are several significant limitations and challenges to consider. Variability in individual responses because of genetic and environmental factors can lead to inconsistent therapeutic outcomes. The complexity of the epigenome, involving the intricate regulation of DNA methylation, histone modifications, and non-coding RNAs, makes it difficult to modulate specific epigenetic markers without unintended effects accurately. Many natural compounds suffer from poor bioavailability, rapid metabolism, and the inability to reach target tissues at effective concentrations. Although generally considered safer than synthetic drugs, natural bioproducts are not completely free from potential side effects, toxicities, or interactions, especially at higher doses or with prolonged use. The lack of standardization and quality control measures for these compounds can affect consistency and reproducibility. Furthermore, the precise mechanisms by which many bioproducts modulate epigenetic pathways are not completely understood. Their complex compositions with the potential for combinatorial effects complicate predicting and controlling the therapeutic impact. Overcoming these challenges through further research, optimized delivery methods, comprehensive clinical trials, and improved standardization is crucial for realizing the full potential of natural epibioproducts in CVD management.

Natural bioproducts with epigenetic properties offer substantial benefits for cardiovascular health management. These compounds provide multi-targeted approaches by simultaneously affecting various epigenetic pathways, including DNA methylation, histone modifications, and non-coding RNA regulation. Their primary advantages include reduced toxicity compared to synthetic drugs, pleiotropic effects addressing multiple aspects of cardiovascular pathophysiology, and potential for long-term preventive use. Polyphenols such as resveratrol and EGCG show promising cardioprotective effects through their ability to modulate DNMT activity and histone acetylation patterns, while curcumin’s potent HDAC inhibitory properties help prevent cardiac hypertrophy and fibrosis. Pharmacoepigenetics, particularly with regard to CVDs, has been underexplored in recent decades. Understanding how external factors influence genomic changes opens new doors for developing more effective epidrugs with fewer side effects than current treatments, potentially reducing the burden of cardiovascular diseases and improving patient quality of life.

Future research directions should prioritize (1) elucidating precise mechanisms of action for natural compounds on specific epigenetic pathways in cardiovascular tissues, (2) developing novel delivery systems to increase bioavailability and tissue targeting, (3) identifying synergistic combinations of natural compounds for increased efficacy, (4) conducting large-scale clinical trials with standardized preparations, and (5) exploring personalized approaches based on individual epigenetic profiles.

It is important to acknowledge that sex is a critical factor in the onset, progression, and prognosis of cardiovascular diseases. Males and females exhibit different risk profiles, with women typically developing CVD approximately 7–10 years later than men and show a higher prevalence of microvascular dysfunction, while men more frequently present with obstructive CAD [226]. Disease progression patterns also differ, with women experiencing higher rates of heart failure with preserved ejection fraction and men showing greater susceptibility to heart failure with reduced ejection fraction [227]. Responses to conventional therapies also vary, with women often experiencing more adverse drug reactions and different pharmacokinetic profiles for medications such as β-blockers and ACE inhibitors [228]. Natural epibioproducts may offer a unique advantage in addressing this sex gap through their ability to modulate epigenetic mechanisms in a sex-specific manner. For instance, miR-34a, miR-155, and miR-125b involved in cardiac remodeling after myocardial infarction show sex-specific expression patterns and could be targeted for tailored epigenetic interventions [226]. Future research should therefore prioritize sex-specific analyses of epigenetic modifications and incorporate sex as a biological variable in the development and testing of natural epibioproducts for cardiovascular applications. This approach may lead to more personalized and effective therapeutic strategies that account for sex-based differences in cardiovascular pathophysiology.

In conclusion, this review highlights the significant potential of natural bioproducts with epigenetic properties in preventing and managing CVDs. These compounds offer promising therapeutic strategies by targeting key epigenetic mechanisms such as DNA methylation, histone modifications, and miRNA regulation. Continuous research and clinical trials are essential to realize the potential of epigenetic therapies. Integrating these emerging therapies into existing treatment frameworks is essential to maximize their efficacy. Current clinical trials and research into natural epibioproducts and their impact on gene expression and methylation patterns are promising. For example, ongoing studies on the cardioprotective effects of polyphenols and their ability to modulate epigenetic markers may translate into more personalized and effective treatment strategies (Table 6). As we move forward, it is important to expedite the translation of these findings into clinical practices, which could revolutionize the management of CVDs.

## Figures and Tables

**Table 2:** Regulation of histone modification in CVDs.

Diseases	Type of Modification	Major Regulator	Target Gene	Mechanism	Reference
Myocardial hypertrophy	Histone methylation	Histone methyltransferase G9a	Histone 3 lysine 9 EZH2	Promotes cardiomyocyte growth by silencing anti-hypertrophic genes	[66]
Vascular calcification	Histone methylation	IL-6/SIL-6R	Histone 3 lysine 9 JMJD2B	Increases osteogenic transdifferentiation of VSMCs	[67]
	Histone methylation	EZH2	TAGLN	Suppresses smooth muscle cell markers during calcification	[68]
	Histone acetylation	SIRT6	Runx2	Inhibits osteogenic transcription factor activation	[69]
	Histone acetylation	HDAC4	Sox9, Runx2, ALP	Regulates osteogenic differentiation of VSMCs	[70]
	Histone acetylation	HDAC9	Runx2	Promotes vascular smooth muscle calcification	[71]
	Histone acetylation	SIRT1	RUNX2, osteocalcin	Prevents osteogenic differentiation in diabetic conditions	[72]
Atherosclerosis	Histone acetylation	SIRT1	eNOS	Activates eNOS, improving vascular function	[22]
	Histone acetylation	HDAC3	eNOS	Reverses aspirin-induced eNOS acetylation	[22]
	Histone acetylation	SIRT1	P65, P300, NF-kB	Suppresses inflammatory signaling in vascular cells	[73]
Myocardial infarction	Histone acetylation	SIRT2	FOXO3A	Triggers nuclear translocation, inducing apoptosis	[74]
	Histone acetylation	SIRT3	Cyclophilin D	Prevents mitochondrial permeability transition pore opening	[75]
	Histone acetylation	HDAC6	Peroxyredoxin 1	Modulates redox status during ischemia/reperfusion	[76]
Heart failure	Histone acetylation	SIRT2	Angiotensin II	Protects against cardiac hypertrophy and remodeling	[77]
	Histone acetylation	SIRT3	GSK3β, SMAD3	Blocks aging-associated cardiac tissue fibrosis	[78]
	Histone acetylation	SIRT4	Angiotensin II	Accelerates pathological cardiac hypertrophy	[79]
	Histone acetylation	SIRT6	P300	Suppresses cardiomyocyte hypertrophy	[80]
	Histone acetylation	SIRT1	NOTCH1	Regulates proliferative signaling in cardiomyocytes	[81]
	Histone acetylation	SIRT7	P53	Increases stress resistance and prevents apoptosis	[82]
Hypertension	Histone acetylation	HDAC6	CSEγ	Regulates cystathionine γ-lyase preventing degradation	[83]
	Histone acetylation	SIRT3	SOD2	Modulates antioxidant enzyme activity	[19]
	Histone acetylation	HDAC1/2	Npr1	Represses natriuretic peptide receptor expression	[84]
	Histone acetylation	HDAC2	KCNJ2K^+^ ion channel	Regulates cardiac action potential duration	[85]

**Table 3:** Regulation of non-coding RNAs in CVDs.

Diseases	Type of Non-Coding RNA	Major Regulator	Target Gene	Mechanism	Reference
Coronary heart disease	miRNA	miRNA-SNP rs41291957	miRNA-143, miRNA-145	Alters vascular smooth muscle cell differentiation	[86]
Atherosclerosis	miRNA	miRNA-1	KLF4	Regulates endothelial cell function and inflammation	[87]
	miRNA	miR-92a	KLF4	Promotes endothelial dysfunction and plaque formation	[88]
	LncRNA	LncRNA Mexis	ABCA1	Modulates cholesterol efflux and lipid metabolism	[89]
	LncRNA	LncRNA NEXN-AS1	TLR-4 oligomer, NF-kB	Mitigates inflammatory response in vascular cells	[90]
Acute myocardial infarction	miRNA	miR-125b	SIRT7	Facilitates cardiac repair by preventing cell death	[91]
	miRNA	miR-21a-5p	PDCD4, PTEN, Peli1, FasL	Mediates cardioprotection by mesenchymal stem cells	[92]
	miRNA	miR-25-3p	E2Z2	Attenuates cardiomyocyte apoptosis	[92]
	miRNA	miR-144	PTEN/AKT	Increases cardiomyocyte survival under hypoxic conditions	[93]
Heart failure	miRNA	miR-425, miR-744	TGF-β	Represents progression of fibrosis and heart failure	[94]
	LncRNA	LncRNA Meg3	MMP2	Regulates cardiac fibrosis and matrix remodeling	[95]
	LncRNA	LncRNA Whisper	Col3a1, Fn1, TGFb2, αSma	Regulates cardiac fibroblast activation and fibrosis	[96]
Vascular calcification	miRNA	miR-30b	MMPs, SOX9	Attenuates phenotypic transformation of VSMCs	[97]
	miRNA	miR-128-3p	Wnt-1, β-catenin, GSK-3β, Bax, Islet1	Accelerates cardiovascular calcification in diabetes	[98]
	miRNA	miRNA-19A-3p	HDAC4	Promotes osteogenic differentiation	[99]
	LncRNA	LncRNATUG1	miRNA-204-5p	Increases osteoblast differentiation via *Runx2*	[100]
	LncRNA	Lrrc75a-as1	SRF, CREB1, STAT3	Regulates VSMC phenotypic switching	[101]
	LncRNA	LncRNA-SNHG29	miR-200b-3p	Inhibits VSMC calcification via α-Klotho/FGFR1/FGF23 axis	[102]
	LncRNA	Bhlhe40 lncRNA-ES3	miR-95-5p, miR-6776-5p, miR-3620-5p, miR-4747-5p	Mediates high glucose-induced VSMC calcification	[103]
Hypertension	miRNA	miR-181a-5p, miR-663	renin	Regulates renin–angiotensin system activity	[104]
	LncRNA	HAS2-AS1	C/EBPβ	Promotes cell migration and inflammation	[105]
	LncRNA	MRAK048635_P1	α-SMA, SM22a, calponin, osteopontin	Regulates VSMC function and phenotypic switching	[106]
Metabolic cardiomyopathy	miRNA	miRNa-494-3p	JunD/PPARα	Promotes myocardial lipid accumulation	[107]
Cardiomyocyte differentiation	LncRNA	Linc1405	Eomes, MesP1	Promotes cardiac mesoderm specification	[108]
Cardiac regeneration	LncRNA	LncRNA CAREL	MiR-296	Regulates cardiac regenerative capacity	[109]
	LncRNA	LncRNA NR_045363, Sirt1 antisense LncRNA	miRNA-216a, Sirt1 mRNA	Promotes cardiomyocyte proliferation	[110,111]
Myocardial infarction	LncRNA	LncRNA Gpr19	Mir-325-5p, Mtfr1	Regulates apoptosis and oxidative stress	[112]
	LncRNA	LncRNA UCA1	Mir-143, MDM2, p53	Protects cardiomyocytes against hypoxia/reoxygenation	[113]
Atrial fibrillation	LncRNA	LncRNA MIAT	miR-133a-3p	Regulates atrial fibrillation and myocardial fibrosis	[114]

**Table 4:** The effects of a selection of bioproducts on DNA methylation for treating CVDs.

Bioproduct	Source	Effects on DNA Methylation	Potential CVD Benefits
Polyphenols	- Green tea (EGCG) - Turmeric (curcumin)	- Inhibits DNMT activity - Decreases DNMT1 expression and global methylation	- May prevent atherosclerosis progression - Anti-inflammatory and improved lipid profiles
Flavonoids	- Soy (genistein) - Fruits/vegetables (quercetin)	- Decreases DNMT1, DNMT3B expression - Inhibits DNMT1 activity	- Reduces inflammation - Improves endothelial function
Resveratrol	- Grapes, berries	- Inhibits DNMT3B	- Reduces inflammation and oxidative stress
Sulforaphane	- Broccoli	- Inhibits DNMT activity	- Reduces global DNA methylation
Genistein	- Soy	- Decreases DNMT1, DNMT3B	- Reduces inflammation
Lycopene	- Tomatoes	- Reduces DNMT1 expression	- Reduces global methylation levels
RCI-1502	- European *S. pilchardus* Walbaum	- Reduces global DNA hypermethylation	- Regulates cholesterol and triglyceride levels in dyslipidemia
Folic Acid	- Dietary supplement	- Increases global DNA methylation	- Potential CVD risk reduction

**Table 5:** miRNAs regulated by bioproducts.

Compound	Induced miRNAs	Inhibited miRNAs
Resveratrol	miR 221 miR 222 miR 15b	miR 126 miR 21 miR 155 miR 34a
Gallic acid	miR 145	miR 21
Garlic	miR126 miR 210	
Curcumin	miR 126	
Kaempferol	miR 21	miR 15b
Quercetin	miR 21	miR 155 miR 199a
Apigenin		miR 15b
Luteolin		miR 21
Ampelopsin		miR 21
Puerarin	miR 21 miR 15b	
Genistein		miR 34a miR 155
Crocin	miR 126 miR 210	miR 34a
Geniposide	miR 145	

**Table 6:** Epidrugs currently submitted or approved for clinical trials for cardiovascular disorders.

Study Title	Study Objectives/Results
The effect of Tongguan Capsule for MicroRNA profiles in Coronary Heart Disease patients (NCT02850627: Interventional; No results posted)	To test the expression of microRNAs related to the syndromes after the intervention of Tongguan capsule.
Air pollution, Epigenetics, and cardiovascular health: a human intervention Trial (NCT01864824: Interventional; No results posted)	To test the effects of methyl-donors on a battery of cardiovascular endpoints highly sensitive to particle pollution.
Nicotinamide Riboside in Systolic Heart Failure (NCT03423342: Interventional; Recruiting)	To determine the safety and tolerability of Nicotinamide Riboside in patients with clinically stable systolic heart failure.
	Nicotinamide Roboside treatment increased whole blood NAD+ levels and changed mitochondrial function. A significant number of patients had abnormal laboratory values and/or adverse events related to treatment.
Mechanistic studies of Nicotinamide Riboside in Human Heart Failure (NCT04528004: Recruiting; Interventional)	To demonstrate the effects of increasing NAD+ levels in heart failure patients.
Nicotinamide Riboside in LVAD recipients (NCT03727646: Interventional)	To test the hypothesis that oral Nicotinamide Riboside supplementation increases myocardial NAD+ levels and improves cardiomyocyte mitochondrial function in individuals with advanced heart failure planned for elective left ventricular assist device (LVAD).
	Nicotinamide Riboside administration enhanced PBMC respiration and reduced proinflammatory cytokine gene expression.

## Data Availability

No new data were created or analyzed in this study. Data sharing is not applicable to this article.
